# Long-Term Outcomes of a Multimodal Day-Clinic Treatment for Chronic Pain under the Conditions of Routine Care

**DOI:** 10.1155/2018/9472104

**Published:** 2018-04-01

**Authors:** Mira A. Preis, Elisabeth Vögtle, Nele Dreyer, Stefanie Seel, Ruth Wagner, Klaus Hanshans, Renate Reyersbach, Christoph Pieh, Andreas Mühlberger, Thomas Probst

**Affiliations:** ^1^Georg-Elias-Müller Institute for Psychology, Georg-August University Göttingen, Göttingen, Lower Saxony, Germany; ^2^Institute for Psychology, Regensburg University, Regensburg, Germany; ^3^Hospital Barmherzige Brüder, Regensburg, Germany; ^4^Department for Psychotherapy and Biopsychosocial Health, Danube University Krems, Krems, Austria

## Abstract

Chronic pain has high prevalence rates and is one of the top causes of years lived with disability. The aim of the present study was to evaluate the long-term effects of a multimodal day-clinic treatment for chronic pain. The sample included 183 chronic pain patients (114 females and 69 males; 53.3 ± 9.8 years) who participated in a four-week multimodal day-clinic treatment for chronic pain. The patients' average current pain intensity (NRS), sensory and affective pain (Pain Perception Scale), and depression and anxiety (HADS) were assessed at pre- and posttreatment, as well as at three follow-ups (one month, six months, and twelve months after completion of the treatment). Multilevel models for discontinuous change were performed to evaluate the change of the outcome variables. Improvements from pretreatment to posttreatment and from pretreatment to all follow-ups emerged for pain intensity (NRS; 0.54 ≤ *d* ≤ 0.74), affective pain (Pain Perception Scale; 0.24 ≤ *d* ≤ 0.47), depression (HADS; 0.38 ≤ *d* ≤ 0.53), and anxiety (HADS; 0.26 ≤ *d* ≤ 0.43) (all *p* < 0.05). Sensory pain as assessed with the Pain Perception Scale did not show any significant change. Patients suffering from chronic pain benefited from the multimodal pain treatment up to twelve months after completion of the treatment.

## 1. Introduction

Chronic pain is a major health care problem. A recent review and meta-analysis including 86 studies found an average prevalence estimate of 31% [1]. Chronic pain is a disabling condition with multidimensional impact on patients, their families, and daily life [2], as well as on work-related variables like loss of employment, early retirement, sick leave, or loss of productivity [3–5]. In the last years, pain constantly is one of the top causes of years lived with disability [6]. Lee et al. [7] reported that self-efficacy, psychological distress, and fear contribute to the process how pain leads to disability.

The interaction between pain and these psychological variables as well as mental disorders like depression and anxiety [8, 9] makes multimodal treatments necessary. Multidisciplinary treatments for chronic pain have been found to be more effective than single-discipline treatments already in 1992 [10] and 2001 [11]. A more recent systematic meta-analysis including 41 randomized controlled trials (RCTs) reported that multidisciplinary treatment for pain is effective to reduce pain and disability and had positive influences on work status compared to physical therapy alone or treatment as usual [12]. The strength of RCTs is their internal validity, but the external validity can be limited [13]. In the area of chronic pain, for example, it has been found that patients “who were randomised were different, in a number of ways, from the entire eligible patient population that was originally identified” (p. 98) [14]. Therefore, there is a broad consensus that interventions should show their *efficacy* in RCTs under controlled conditions and their *effectiveness* under clinically representative conditions [15]. With regard to multimodal therapy for patients with chronic pain, there is some evidence for its effectiveness under clinically representative conditions as well [16–27].

Despite these effects of pain management treatments in controlled and clinically representative contexts, Wilson [28] stated in a recent critical review that more research on the long-term outcomes and the sustaining effects of such programs for chronic pain is necessary.

To further evaluate the long-term effectiveness of multidisciplinary treatments for chronic pain under the conditions of routine care, the present study investigated the effects of a multimodal day-clinic treatment for chronic pain on aspects of pain (pain intensity and affective and sensory pain) and on depression and anxiety. We hypothesized that the multidisciplinary treatment is effective up to 12 months after treatment.

## 2. Materials and Methods

### 2.1. Sample

The sample consisted of 183 patients (114 females and 69 males; mean age of 53.3 ± 9.8 years) who participated in the multimodal day-clinic treatment for chronic pain at the Hospital Barmherzige Brüder Regensburg (Germany) from 2010 to 2013. All patients fulfilled the criteria for the ICD-10 diagnosis of a chronic pain disorder with somatic and psychological factors (F45.41). 75.4 percent of the patients fulfilled the criteria for at least one other psychiatric disorder, with depression (57.9 percent) and anxiety disorders (22.4 percent) being the most frequently diagnosed comorbidities. The four most frequent medical diagnoses according to ICD-10 were dorsalgia (M54), other disorders of the muscle (M62), other headache syndromes (G44), and other unspecified dorsopathies (M53). The diagnoses were made by the clinic team. With regard to Von Korffs' chronic pain grades [29] that use pain intensity and pain-related disability to grade pain into four hierarchical classes (grade I, low disability-low intensity; grade II, low disability-high intensity; grade III, high disability-moderately limiting; and grade IV, high disability-severely limiting), the majority of the patients showed high disability and moderate-to-high limiting with four percent of the patients classified as grade 1, 16.2 percent as grade II, 29.5 percent as grade III, and 50.3 percent as grade IV. All patients provided written informed consent before their participation.

### 2.2. Psychometric Instruments

The following self-rating instruments were given at pre- and posttreatment, as well as one month, six months, and twelve months after completion of the treatment. At the three-month follow-up assessment, the clinic sent a paper and pencil version of the questionnaires by post to the patients. After completing the questionnaires, the patients sent them back to the clinic.

#### 2.2.1. Pain Intensity

The participants rated the average current pain intensity on a Numerical Rating Scale (NRS) from 0 (*no pain*) to 10 (*worst imaginable pain*).

#### 2.2.2. Pain Perception Scale

The Pain Perception Scale (SES [30]) includes 24 items that describe the affective (14 items, e.g., *nagging* and *unbearable*) and sensory qualities of pain (10 items, e.g., *hot* and *pulsative*). Each item is rated on a 4-point scale from 0 (*not appropriate*) to 3 (*appropriate*). Both scales show good internal consistencies, with Cronbach's *α* = 0.92 for the affective scale and *α* = 0.81 for the sensory scale [30].

#### 2.2.3. Depression and Anxiety

Designed for clinical populations suffering from somatic symptoms, the German version of the Hospital Anxiety and Depression Scale (HADS) was used to assess anxiety and depression in the past week [31, 32]. The HADS includes 14 items, of which seven assess anxiety (e.g., *I feel tense or overstrung*), whereas the other seven items measure depression (e.g., *I am happy*). All items are rated on a 4-point scale. Both scales reach satisfactory internal consistencies (Cronbach's *α* = 0.80 for both depression and anxiety [31]).

### 2.3. Procedure/Treatment

**Figure 1:**
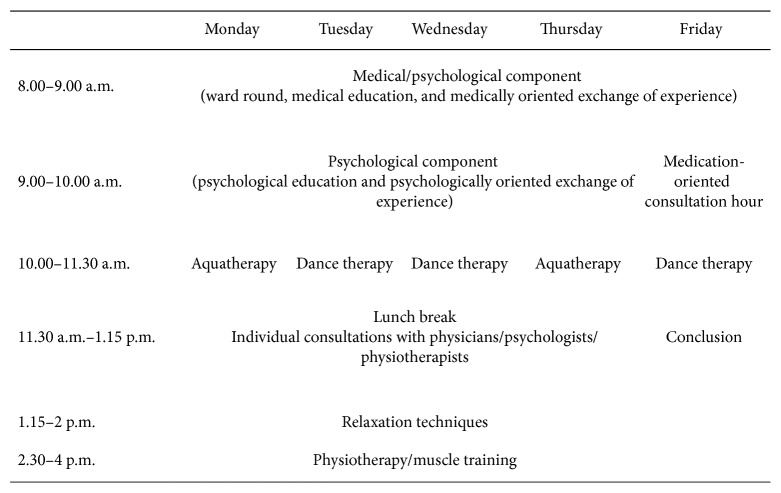
Weekly schedule of the multimodal treatment program.

At the Hospital Barmherzige Brüder Regensburg (Germany), an interdisciplinary team of psychologists, physicians, physical therapists, occupational therapists, and social workers carried out the 4-week multimodal day-clinic treatment for chronic pain. The treatment is based on cognitive-behaviour therapy for pain “*Marburger Schmerzbewältigungsprogramm*” [33]. Each of the 4 treatment weeks proceeded according to the same schedule that lasted from Monday to Friday (8.00 a.m. to 4.00 p.m.; on Fridays: 8.00 a.m. to 1.15 p.m.) and was composed of different treatment elements including medical and psychological modules as well as physical therapy (see Figure 1 for an overview of the weekly schedule). Psychological group sessions included psychoeducation and comprehension of the biopsychosocial pain model, directing the attention towards positive experiences, as well as relaxation techniques. The maximal group size was eight patients and continued throughout the program with the same patients (closed groups).

### 2.4. Statistics

SPSS 24 was used for the statistical analyses. Multilevel models for discontinuous change were performed to evaluate the progress of the outcome variables (pain intensity, affective pain, sensory pain, depression, and anxiety) between the five assessment points. According to Göllner et al. [34], four contrast variables were created to investigate the course of the outcomes from (1) pretreatment to the end of treatment, (2) pretreatment to 1-month follow-up, (3) pretreatment to 6-month follow-up, and (4) pretreatment to 12-month follow-up. All multilevel models were performed with the full maximum likelihood estimation, and an unstructured variance-covariance matrix was selected. The multilevel models included two levels: assessments as level 1 and patients as level 2. The statistical tests were performed two-tailed, and the statistical significance value was set to *p* < 0.05. Moreover, effect sizes (*d*) were calculated with the means and standard deviations at the five assessment points as follows: (*M*_pretreatment_ – *M*_posttreatment_or_follow-up_)/SD_pretreatment_. Effect sizes were calculated with the values given in Table 1. Furthermore, differences between the pretreatment values of the NRS and the posttreatment values of the NRS and differences between the pretreatment NRS scores and the follow-up NRS scores were computed. The differences were calculated only for those patients with available NRS scores at both assessment points to compute differences. Patients with an improvement ≥2 points on the NRS were classified as patients with clinically important improvements [35].

## 3. Results

### 3.1. Dropout

Dropout rates differed between the measures. For the Numeric Rating Scale (NRS) that assessed the average current pain intensity, response rates ranged from 100% at pretreatment to 67.2% at 12-month follow-up. Response rates concerning the Pain Perception Scale (SES [30]) that measured affective and sensory pain qualities ranged from 91.8% at pretreatment to 53% at 12-month follow-up. Response rates regarding symptoms of anxiety and depression that were recorded by means of the Hospital Anxiety and Depression Scale (HADS [31]) reached from 98.4% at pretreatment to 64.5% at 12-month follow-up. For a full overview of the dropout rates, see Table 1.

### 3.2. Treatment Effects on Pain Characteristics

#### 3.2.1. Pain Intensity

The estimates of the multilevel model with pain intensity (numeric rating scale) as outcome are presented in Table 2. Reductions of pain intensity became statistically significant from pretreatment to the end of treatment (*d*=0.59;  *p* < 0.001), from pretreatment to 1-month follow-up (*d*=0.74;  *p* < 0.001), from pretreatment to 6-month follow-up (*d*=0.54;  *p* < 0.001), and from pretreatment to 1-year follow-up (*d*=0.62;  *p* < 0.001).

In addition, we calculated the percentage of patients who reached a reduction of at least two points on the NRS from pretreatment to the other assessment points. A pain reduction of 2 points or more on the NRS was reached by 34.1% at the end of the treatment, by 45.2% at 1-month follow-up, by 36.8% at 6-month follow-up, and by 36.6% at 1-year follow-up. The percentages are in relation to the sample of patients with NRS scores at both assessment points.

#### 3.2.2. Affective Pain

Table 3 summarizes the results of the multilevel model with affective pain (Affective scale of the Pain Perception Scale [30]) as outcome. It can be seen that the affective component of pain was significantly improved from pretreatment to the end of treatment (*d*=0.29;  *p* < 0.001), from pretreatment to 1-month follow-up (*d*=0.47;  *p* < 0.001), from pretreatment to 6-month follow-up (*d*=0.24;  *p*=0.010), and from pretreatment to 1-year follow-up (*d*=0.44;  *p*=0.001).

#### 3.2.3. Sensory Pain

The results of the multilevel model with sensory pain (Sensory scale of the Pain Perception Scale [30]) as outcome are shown in Table 4. No statistically significant changes emerged from pretreatment to the end of treatment (*d*=0.08;  *p*=0.231), from pretreatment to 1-month follow-up (*d*=0.10;  *p*=0.195), from pretreatment to 6-month follow-up (*d*=0.03;  *p*=0.856), and from pretreatment to 1-year follow-up (*d*=0.07;  *p*=0.142).

### 3.3. Treatment Effects on Psychological Variables

#### 3.3.1. Depression

Table 5 includes the results of the multilevel model with depression (Depression scale of the Hospital Anxiety and Depression Scale [31]) as outcome. All comparisons reached statistical significance: depression improved from pretreatment to the end of treatment (*d*=0.53;  *p* < 0.001), from pretreatment to 1-month follow-up (*d*=0.46;  *p* < 0.001), from pretreatment to 6-month follow-up (*d*=0.45;  *p* < 0.001), and from pretreatment to 1-year follow-up (*d*=0.38;  *p*=0.003).

#### 3.3.2. Anxiety

Results for the multilevel model with anxiety (Anxiety scale of the Hospital Anxiety and Depression Scale [31]) as outcome are given in Table 6. The significant results indicate that anxiety decreased from pretreatment to the end of treatment (*d*=0.40;  *p* < 0.001), from pretreatment to 1-month follow-up (*d*=0.43;  *p* < 0.001), from pretreatment to 6-month follow-up (*d*=0.26;  *p*=0.004), and from pretreatment to 1-year follow-up (*d*=0.34;  *p*=0.002).

## 4. Discussion

The aim of the present study was to evaluate the long-term effects of a multimodal day-clinic treatment for chronic pain on pain characteristics (pain intensity and sensory and affective pain) and associated psychological aspects (depression and anxiety). The 4-week pain treatment significantly reduced the patients' pain intensity, depression, and anxiety, improved the appraisal of affective qualities of pain, and remained stable over a period of 12 months after completion of the treatment. There was no effect on the Sensory Pain scale of the Pain Perception Scale.

In the following paragraphs, we embed our results in the existing literature on the effectiveness of multimodal treatments for chronic pain under clinically representative conditions. When interpreting these benchmark comparisons, it is important to consider the following aspects as possible reasons for discrepant results. Different treatment durations (4 weeks in the present study and, e.g., in [26] versus 5 weeks in [24, 25]), different treatment settings (e.g., day-clinic treatment in the present study and, e.g., in [24, 25] versus inpatient treatment in [17]), and different dropout rates (e.g., 80% [27] or 59% [17] dropout at 1-year follow-up versus <50% dropout at 1-year follow-up in the present study).

The patients' pain intensity improved from pre- to posttreatment with a medium effect size of *d*=0.59. This is comparable to the medium effect sizes of *d*=0.64 found by Hampel et al. [21], *d*=0.6 reported by Moradi et al. [22], and *d*=0.51 found by Ruscheweyh et al. [26]. However, Pieh et al. [24] and Pöhlmann et al. [25] reported larger effect sizes of *d*=1.0 and *d*=0.69 − 0.98, respectively, concerning the reduction of the pain intensity at the end of a 5-week multimodal day-clinic treatment for patients with chronic pain. Borys et al. [17] reported a higher effect size of *d*=0.74 at the end of 3-week inpatient treatment. Over a period of six and twelve months, pain intensity was improved in our study, which indicates that the patients could implement their acquired knowledge of the treatment in their everyday life and profit in the long term. With regard to Schütze et al. [27], the effect size at the six-month follow-up is comparable (*d*=0.54 versus *d*=0.55), whereas the twelve-month follow-up effect size is smaller in the current study (*d*=0.62 versus *d*=0.95 for 20% of the patients taking part in the follow-up in [27]). While Moradi et al. [22] (*d* = 0.7) and Pöhlmann et al. [25] (0.69 − 0.98) reported higher effect sizes at 6-month follow-up Ruscheweyh et al. [26] found a smaller long-term effect of *d*=0.33 at that time as did Hampel et al. [21] (*d*=0.37). The effect size for pain intensity at 12-month follow-up in the current study (*d*=0.62) was higher than the one found by Borys et al. [17] (*d*=0.20). With regard to Farrar et al. [35] who proposed that a pain reduction of approximately two points on the NRS represents a clinically important difference, 34 percent of the patients showed an improvement of clinical importance at the end of the treatment. With approximately 37 percent, this ratio of clinically important improvement was stable to one year after the treatment.

There was a positive effect on the appraisal of affective pain qualities. Although the effect directly after the treatment (*d*=0.29) was smaller than that in previous studies (e.g., Pieh et al. [24]: *d*=0.7; Hampel et al. [21]: *d*=0.55) as was the effect at 6-month follow-up (*d*=0.24 vs. *d*=0.37 in Hampel et al. [21]), the appraisal of affective pain qualities improved further over time and reached an effect size of *d*=0.44 at twelve months after completion of the treatment. It emphasizes that the treatment may have initiated a process of reappraisal that continued and intensified even further after completion.

In contrast to Pieh et al. [24], the current treatment caused no significant changes regarding the Sensory scale of the Pain Perception Scale. In the study by Hampel et al. [21], there was a significant but small improvement (*d*=0.25) that diminished after six months. Hammes et al. [36] concluded that treatment (acupuncture in their study) in patients with high chronic pain preferentially improves the affective dimension of pain. With reference to Von Korffs' chronic pain grades [29], the majority of the patients in this study can be described as highly chronic.

The reduction of depressive symptoms at the end of the treatment (*d*=0.53) was comparable to some previous studies (e.g., Pieh et al. [24]: *d*=0.54; Hampel et al. [21]: *d*=0.57; Ruscheweyh et al. [26]: *d*=0.42) but smaller than that observed by Borys et al. [17] (*d*=0.77), Moradi et al. [22] (*d*=0.7), and Pöhlmann et al. [25] (*d*=0.80). In contrast to Borys et al. [17], Hampel et al. [21], and Ruscheweyh et al. [26] who reported that depression rates at follow-up (twelve months and six months, resp.) were no longer different from pretreatment, the reduction in symptoms of depression was significant at twelve-month follow-up in the current study (*d*=0.38). The effect size for depression at 6-month follow-up (*d*=0.45), however, was lower than the effect sizes reported by Moradi et al. [22] and Pöhlmann et al. [25] (both  *d*=0.7). Compared with Schütze et al. [27] (*d*=0.10–0.20) and Ruscheweyh et al. [26] (*d*=0.26), the improvement at posttreatment regarding symptoms of anxiety represents a slightly higher effect (*d*=0.40). Compared to Borys et al. [17] (*d*=0.55), however, the effect on anxiety at discharge was lower. At 12-month follow-up, anxiety improved (*d*=0.34) slightly more than that in Borys et al. [17] (*d*=0.22). Anxiety at 12-month follow-up was not different from baseline in the study by Ruscheweyh et al. [26]. The effect sizes for anxiety at the 6-month and 12-month follow-ups were between 0.2 and 0.3 in the study by Schütze et al. [27]. Corresponding to a previous study that documents a high prevalence of depression in chronic pain patients attending treatment [37], depression was the most prevalent comorbidity in the current sample.

### 4.1. Limitations and Strengths

The data of the current study were collected in a naturalistic setting. This enhances the external validity of the results. But as the study was neither randomized nor controlled, the internal validity of the results is limited. Therefore, we cannot exclude confounders like time effects as possible causes for the found improvements. Yet, a 4-year follow-up study on the course of chronic pain in the community reported that chronic pain shows low recovery rates [38]. Nevertheless, aspects within and outside the treatment could have influenced our effects, and we cannot conclude what component of the treatment was effective, which components of the treatment contributed the most, or whether a single modal treatment would have had similar effects. However, it should be kept in mind that RCTs and component studies are difficult to realize under the conditions of routine care. Moreover, no information regarding potential treatments during the 12-month follow-up was available. A strength of the current study is the relatively low dropout rate (<50% for each questionnaire at 12-month follow-up). Moreover, the use of multilevel models for discontinuous change in order to evaluate the progress of the outcome variables is a strength of the current study as it is more flexible than the most commonly used repeated measures analysis of variance (ANOVA). Further strengths comprise the several follow-up assessments and the use of psychometrically sound questionnaires. Another limitation is that attendance rates were not recorded so that we could not analyze how many patients received the complete treatment and how the attendance might be correlated with the outcome.

## 5. Conclusions

As chronic pain is most probably caused by an interaction of biopsychosocial factors, multimodal pain treatment programs seem to provide the most effective therapy. The current study supports the notion that chronic pain patients benefit from multimodal treatments under the conditions of routine care in the long term.

## Figures and Tables

**Table 1:** Sample size (*n*), means (*M*), and standard deviations (SD) of the measures per assessment point.

	Pretreatment			End of treatment			1-month follow-up			6-month follow-up			12-month follow-up		
	*n*	*M*	SD	*n*	*M*	SD	*n*	*M*	SD	*n*	*M*	SD	*n*	*M*	SD
NRS	183	6.51	1.66	176	5.53	1.85	168	5.28	1.88	152	5.61	1.91	123	5.48	1.95
SES: affective	168	61.04	24.79	153	53.79	25.54	145	49.39	27.02	139	55.11	29.29	97	50.17	27.61
SES: sensory	168	66.90	26.48	152	64.65	26.96	145	64.37	26.98	138	66.20	28.18	96	65.15	26.75
HADS: depression	180	10.34	4.20	176	8.13	4.59	168	8.42	4.76	155	8.45	4.94	122	8.76	5.03
HADS: anxiety	180	10.49	4.24	176	8.81	4.34	168	8.66	4.49	156	9.39	4.67	118	9.05	4.05

*Note*. NRS: Numeric Rating Scale; SES: Pain Perception Scale; HADS: Hospital Anxiety and Depression Scale.

**Table 2:** Results of the multilevel model for discontinuous change with pain intensity as outcome (*N*=183).

Parameter	Estimate	SE	df	T-statistic	*p*
Intercept (pretreatment)	6.51	0.12	183.00	53.22	<0.001
Change from pretreatment to the end of treatment	−0.97	0.12	178.66	−7.83	<0.001
Change from pretreatment to 1-month follow-up	−1.18	0.14	165.87	−8.58	<0.001
Change from pretreatment to 6-month follow-up	−0.85	0.15	168.24	−5.74	<0.001
Change from pretreatment to 1-year follow-up	−0.99	0.16	159.29	−6.12	<0.001

**Table 3:** Results of the multilevel model for discontinuous change with affective pain as outcome (*N*=174).

Parameter	Estimate	SE	df	T-statistic	*p*
Intercept (pretreatment)	60.93	1.90	170.14	32.06	<0.001
Change from pretreatment to the end of treatment	−7.88	1.58	159.14	−4.98	<0.001
Change from pretreatment to 1-month follow-up	−12.08	1.96	157.33	−6.17	<0.001
Change from pretreatment to 6-month follow-up	−5.85	2.23	151.13	−2.63	0.010
Change from pretreatment to 1-year follow-up	−7.96	2.30	148.83	−3.46	0.001

**Table 4:** Results of the multilevel model for discontinuous change with sensory pain as outcome (*N*=174).

Parameter	Estimate	SE	df	T-statistic	*p*
Intercept (pretreatment)	66.58	2.03	170.00	32.77	<0.001
Change from pretreatment to the end of treatment	−2.24	1.86	159.09	−1.20	0.231
Change from pretreatment to 1-month follow-up	−2.26	1.74	158.28	−1.30	0.195
Change from pretreatment to 6-month follow-up	0.36	1.95	150.76	0.18	0.856
Change from pretreatment to 1-year follow-up	2.89	1.96	134.09	1.48	0.142

**Table 5:** Results of the multilevel model for discontinuous change with depression as outcome (*N*=183).

Parameter	Estimate	SE	df	T-statistic	*p*
Intercept (pretreatment)	10.37	0.31	182.04	33.32	<0.001
Change from pretreatment to the end of treatment	−2.14	0.24	175.39	−8.87	<0.001
Change from pretreatment to 1-month follow-up	−1.73	0.25	167.85	−6.98	<0.001
Change from pretreatment to 6-month follow-up	−1.54	0.30	157.07	−5.12	<0.001
Change from pretreatment to 1-year follow-up	−1.05	0.35	148.07	−2.98	0.003

**Table 6:** Results of the multilevel model for discontinuous change with anxiety as outcome (*N* = 182).

Parameter	Estimate	SE	df	T-statistic	*p*
Intercept (pretreatment)	10.52	0.31	181.62	33.52	<0.001
Change from pretreatment to the end of treatment	−1.69	0.24	174.40	−6.98	<0.001
Change from pretreatment to 1-month follow-up	−1.73	0.26	171.19	−6.60	<0.001
Change from pretreatment to 6-month follow-up	−0.92	0.32	155.92	−2.91	0.004
Change from pretreatment to 1-year follow-up	−0.94	0.30	151.28	−3.09	0.002
